# *In vitro* rejuvenation of brain mitochondria by the inhibition of actin polymerization

**DOI:** 10.1038/s41598-018-34006-5

**Published:** 2018-10-22

**Authors:** Kazuhide Takahashi, Yuri Miura, Ikuroh Ohsawa, Takuji Shirasawa, Mayumi Takahashi

**Affiliations:** 1 Biological Process of Aging, Tokyo Metropolitan Institute of Gerontology, Itabashi, Tokyo 173-0015 Japan; 2 Proteome Research, Tokyo Metropolitan Institute of Gerontology, Itabashi, Tokyo 173-0015 Japan; 3 Shirasawa Anti-Aging Medical Institute, Bunkyo, Tokyo 113-0033 Japan

## Abstract

The oxygen consumption rate (OCR) and cytochrome c oxidase (CcO) activity of respiratory complex IV (CIV) in brain mitochondria significantly decline in middle-aged male mice compared to younger male mice. To explore the mechanisms underlying the regulation of brain mitochondrial function, we examined CIV-associated proteins, and identified actin inside the isolated brain mitochondria. Inhibiting actin polymerization using cytochalasin B (CB) significantly enhanced the OCR and CcO activity of CIV in the mitochondria. These changes were accompanied by a significant reduction in the amount of CIV-bound cytochrome c (cyt c). Actin was also associated with respiratory complex III (CIII); however, the amount of CIII-bound cyt c increased significantly after treatment of the mitochondria with CB. In contrast, no significant alteration in the assembly or the CcO activity of CIV in CIV-containing supercomplexes or CIV monomers was induced by CB. These results suggest that mitochondrial actin plays a crucial role in the regulation of the CcO activity and OCR of CIV with modification of the retention of cyt c between CIV and CIII.

## Introduction

Mitochondrial function extends far beyond that of energy generation, because accumulating evidence has suggested a causative link between mitochondrial dysfunction and major phenotypes associated with aging^1^ and many neurodegenerative diseases^2,3^. We recently found that the earliest decline in mitochondrial function, which was assessed by measuring the mitochondrial oxygen consumption rate (OCR) of respiratory complex IV (CIV), appeared in the brains of 12- to 15-month-old (denoted as middle-aged) male mice during normal aging^4,5^. The reduced brain mitochondrial OCR in the middle-aged mice is accompanied by motor impairment, including reduced exploratory and voluntary motor activities, and an increased accumulation of phosphorylated α-synuclein in the motor cortex^5^. These behavioral and histological alterations that occur during normal aging are similar to the characteristics of one of the most common neurodegenerative diseases, Parkinson’s disease (PD)^6,7^. Moreover, the reduced OCR in brain mitochondria isolated from middle-aged mice is accompanied by reduced cytochrome c oxidase (CcO) activity of CIV^8^. CcO of CIV is the final enzyme in the respiratory electron transport chain, and CIV receives an electron from each of four cytochrome c (cyt c) molecules and transfers the electrons to oxygen molecules. These results demonstrating the age-dependent declines in brain mitochondrial OCR and CcO activity of CIV suggest that CIV is a key player in the regulation of brain mitochondrial function during normal aging.

In this report, we analyzed the CIV-interacting proteins that affect the mitochondrial OCR of the brains isolated from middle-aged male mice. Immunoprecipitation, followed by mass spectrometry, and immunofluorescence analysis indicated that actin was coprecipitated with CIV and localized inside the brain mitochondria. Inhibiting actin polymerization by incubating the isolated brain mitochondria with cytochalasin B (CB) resulted in a significant enhancement of the mitochondrial OCR and CcO activity of CIV. Concomitantly, the amount of CIV-bound cytochrome c (cyt c) was significantly reduced by treatment of the mitochondria with CB. Actin was also coprecipitated with respiratory complex III (CIII); however, the amount of CIII-bound cyt c was significantly increased after the treatment of mitochondria with CB. Regarding the assembly of supercomplexes (SCs)^9,10^, which contain CIV, no significant alterations were induced by inhibiting actin polymerization with CB. These results indicate a novel function of mitochondrial actin in the regulation of brain mitochondrial function.

## Results

### Actin associates with CIV within brain mitochondria

**Figure 1:**
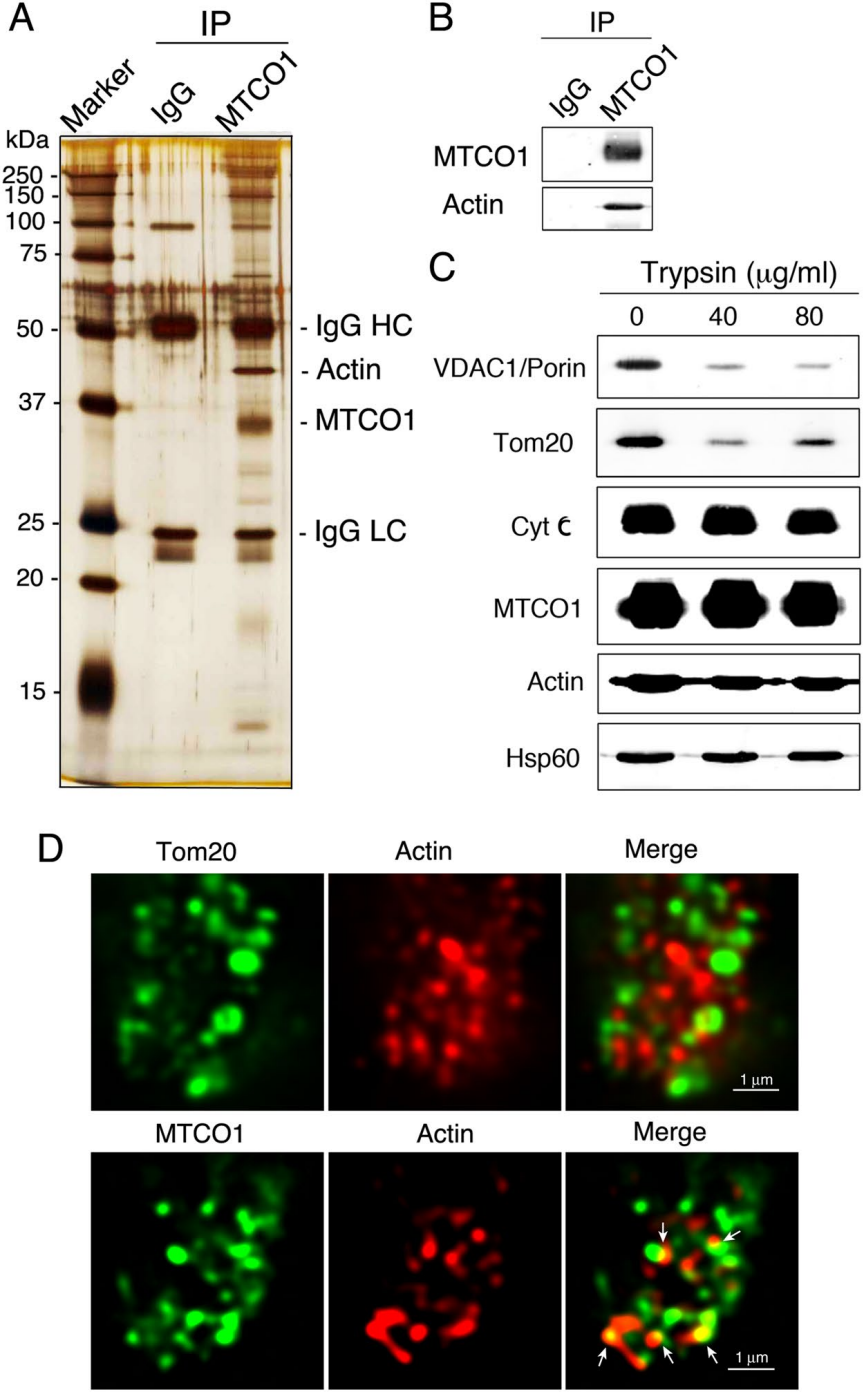
Actin is associated with the CIV subunit MTCO1 and localizes inside brain mitochondria. (**A**) Silver staining of gels after SDS-PAGE of the immunoprecipitates from brain mitochondria isolated from middle-aged male mice with normal IgG and anti-MTCO1 antibody. (**B**) Immunoblot analysis of the immunoprecipitates with normal IgG and anti-MTCO1 antibody using antibodies against MTCO1 and actin. (**C**) Isolated brain mitochondria were digested with trypsin at 37 °C for 30 min, followed by immunoblotting with antibodies against the indicated proteins. (**D**) Confocal images of isolated brain mitochondria after immunofluorescence staining with antibodies against Tom20, actin, and MTCO1. Arrows indicate the contact sites of actin with MTCO1.

An age-dependent decline in the OCR of CIV^4,5^ is accompanied by a reduction in the CcO activity of CIV and L-Opa1 binding to CIV^8^. To further clarify the mechanism underlying the regulation of the OCR, a CIV subunit, MTCO1, was immunoprecipitated from brain mitochondria isolated from 15- to 16-month-old (denoted as middle-aged) male mice, and the CIV-associated proteins were analyzed. Following SDS-PAGE, silver staining of the immunoprecipitates with anti-MTCO1 antibody demonstrated that several proteins were coprecipitated with MTCO1 (Fig. 1A). Among these proteins, the 42 kDa protein was identified as actin (Swiss-Prot, P60710) by mass spectrometry, having a protein score higher than 60 and more than 50 tryptic peptides with an ion score above 95% confidence (Fig. 1A and Table S1). The coprecipitation of actin with MTCO1 was confirmed by immunoblot analysis using specific antibodies against MTCO1 and actin (Fig. 1B).

To maintain normal mitochondrial function, mitochondria undergo fission and fusion^11^. One current model of mitochondrial fission proposes that constriction is initiated by the polymerization of actin microfilaments at the outer surface of mitochondria^12–17^. To eliminate actin outside of the mitochondria, brain mitochondria isolated from middle-aged mice were subjected to progressive proteolysis with serial concentrations of trypsin^18,19^. Treatment of the isolated mitochondria with trypsin removed 80 to 90% of the outer mitochondrial membrane (OMM) proteins VDAC1/porin and Tom 20 (Fig. 1C). In contrast, 80 to 90% of the intermembrane space (IMS) protein cyt c, inner mitochondrial membrane (IMM) protein MTCO1, mitochondrial matrix protein Hsp60, and actin, remained intact, as demonstrated by immunoblot analysis after the digestion of isolated mitochondria with trypsin (Fig. 1C). Under these extreme experimental conditions, the proteolytic activity was strong enough to digest most of the OMM proteins, and slightly digest the IMS protein, IMM protein, mitochondrial matrix protein, and actin.

To visualize the mitochondrial localization of actin and its interaction with MTCO1, immunofluorescence staining was applied to the isolated brain mitochondria. Immunofluorescence staining with antibodies against actin and the OMM protein Tom20 revealed that actin filaments were localized inside the OMM of the brain mitochondria (Fig. 1D, upper panels). Confocal images of fluorescence staining with antibodies against actin and the IMM protein MTCO1 indicated that a portion of the actin filaments contacted MTCO1 within the mitochondria (Fig. 1D, arrows in lower panels). These results indicated that actin filaments are localized inside the brain mitochondria isolated from middle-aged male mice and that a portion of the actin filaments are associated with the IMM protein MTCO1, a subunit of CIV.

### Inhibiting actin polymerization enhances the OCR

**Figure 2:**
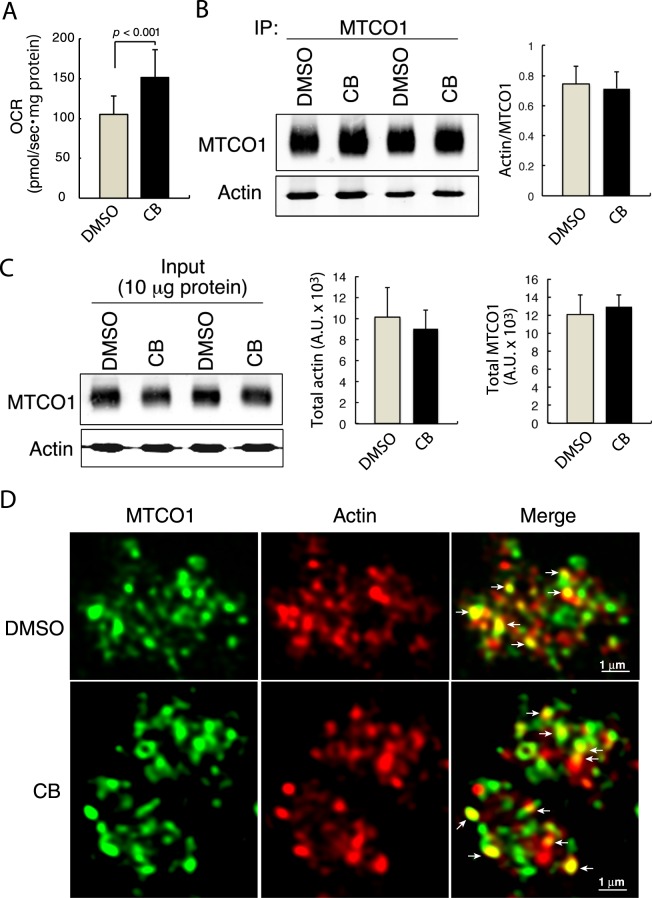
Inhibiting actin polymerization enhances the OCR, with no significant alterations in the association of actin with MTCO1. (**A**) Measurement of the OCR of isolated brain mitochondria during incubation with DMSO or CB with high-resolution respirometry (n = 14, mean ± S.D.). *p* < 0.001. (**B**) Isolated brain mitochondria were incubated with DMSO or CB for 30 min, immunoprecipitated with anti-MTCO1 antibody, and immunoblotted for MTCO1 and actin. The ratio of coprecipitated-actin to MTCO1 was determined (n = 4, mean ± S.D.). (**C**) After incubation of the isolated brain mitochondria with DMSO or CB for 30 min, whole mitochondrial lysates were subjected to SDS-PAGE and immunoblotted for MTCO1 and actin. The total amount of MTCO1 and actin in arbitrary units (A.U.) were compared between samples treated with DMSO and CB (n = 6, mean ± S.D.). (**D**) Confocal images of immunofluorescence staining with anti-MTCO1 (green) and anti-actin (red) antibodies after the treatment of isolated brain mitochondria with DMSO or CB for 30 min. Arrows indicate the contact sites of actin with MTCO1.

To examine whether MTCO1-associated actin in brain mitochondria isolated from middle-aged mice plays a crucial role in the regulation of mitochondrial function, we first measured the OCR when actin polymerization was inhibited by cytochalasin B (CB), a specific inhibitor of actin polymerization^20^. High-resolution respirometry demonstrated that the OCR in the isolated mitochondria was significantly enhanced by treatment with CB (Fig. 2A), indicating enhancement of the OCR by inhibition of actin polymerization. After incubation of the brain mitochondria with CB, immunoblot analysis using antibodies against MTCO1 and actin revealed that the amount of actin that was coprecipitated with MTCO1 remained unchanged (Fig. 2B). In addition, the total amounts of both actin and MTCO1 were comparable between the control and CB-treated brain mitochondria (Fig. 2C). Confocal images of the immunofluorescence staining of actin and MTCO1 revealed that a portion of the actin filaments contacted MTCO1 in both DMSO- and CB-treated mitochondria (Fig. 2D).

### Inhibiting actin polymerization reduces the amount of CIV-bound cyt c

**Figure 3:**
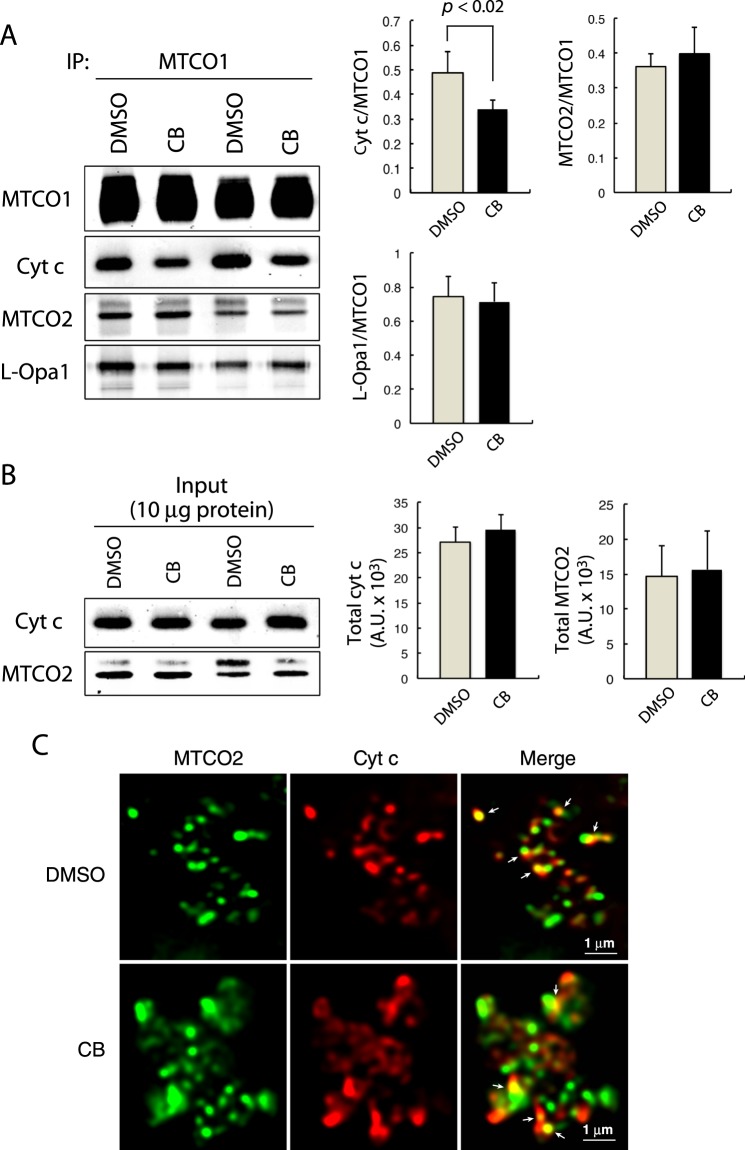
Inhibiting actin polymerization reduces the amount of CIV-bound cyt c. (**A**) Immunoblot analysis of the immunoprecipitates with anti-MTCO1 antibody from DMSO- and CB-treated mitochondria, followed by immunoblotting with antibodies against the indicated proteins. The relative amounts of the MTCO1-associated proteins to MTCO1 were determined (n = 6, mean ± S.D.). *p* < 0.02. (**B**) Immunoblot analysis for the total mitochondrial proteins indicated in DMSO- or CB-treated brain mitochondria (n = 8, mean ± S.D.). (**C**) Isolated brain mitochondria were treated with DMSO or CB and immunostained with anti-MTCO2 (green) and anti-cyt c (red) antibodies. Arrows indicate the contact sites of cyt c with MTCO2.

To clarify the reason for enhancement of the OCR in middle-aged brain mitochondria caused by inhibiting actin polymerization with CB, CIV-associated proteins other than actin within the brain mitochondria were analyzed by immunoblotting. Because CIV has CcO activity and oxidizes cyt c, we first examined the binding of cyt c to MTCO1. Immunoblot analysis of the MTCO1 immunoprecipitates revealed that cyt c was coprecipitated with MTCO1, and the amount of MTCO1-bound cyt c was significantly reduced by treatment with CB (Fig. 3A). Cyt c binds to CIV mainly through MTCO2, a subunit of CIV^21,22^. Therefore, we analyzed the amount of MTCO2 that was coprecipitated with MTCO1 to exclude the possibility that the reduced amount of MTCO1-bound cyt c is due to reduced MTCO2 in CIV. Figure 3A shows that the relative amount of MTCO1-associated MTCO2 to MTCO1 was comparable between mock-treated and CB-treated mitochondria (Fig. 3A). This result suggests that the reduced MTCO1-bound cyt c was not due to the reduced amount of MTCO2 in CIV by resulting from the CB treatment. In contrast to the significant reduction in the amount of MTCO1-bound cyt c by resulting from the CB treatment (Fig. 3A), the total amounts of both cyt c and MTCO2 in the isolated brain mitochondria remained unchanged after treatment with CB (Fig. 3B).

Restoration of the OCR in middle-aged brain mitochondria by CoQ_10_ is accompanied by the increased binding of L-Opa1 to CIV^8^. To examine the difference between CoQ and CB in the mechanism underlying the OCR enhancement *in vitro*, the association of L-Opa1 with MTCO1 was examined. Immunoblot analysis demonstrated that the amount of L-Opa1, which was coprecipitated with MTCO1, remained unchanged after treatment of the isolated brain mitochondria with CB (Fig. 3A). This result suggests that the CB-induced enhancement of the OCR in the isolated brain mitochondria was not dependent on an increased binding of L-Opa1 to MTCO1.

Regarding the mitochondrial localization of cyt c and MTCO2, immunofluorescence staining revealed that a portion of cyt c contacted MTCO2 in control DMSO-treated mitochondria, and the occasional contacts appeared unchanged in CB-treated mitochondria (Fig. 3C).

### Inhibiting actin polymerization increases the amount of CIII-bound cyt c

**Figure 4:**
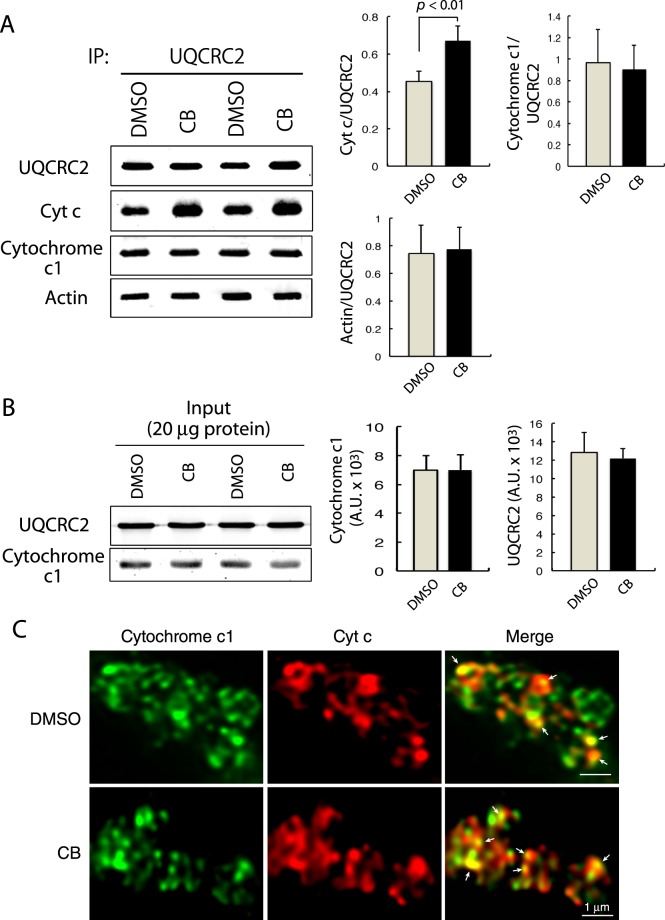
Inhibiting actin polymerization increases the amount of CIII-bound cyt c. (**A**) Isolated brain mitochondria treated with DMSO or CB were lysed and immunoprecipitated with anti-UQCRC2 antibody, followed by immunoblotting for the indicated proteins. The relative amounts of coprecipitated proteins to UQCRC2 were determined (n = 8, mean ± S.D.). *p* < 0.01. (**B**) Whole lysates of DMSO- or CB-treated mitochondria were immunoblotted with antibodies against UQCRC2 and cytochrome c_1_ (n = 6, mean ± S.D.). (**C**) Confocal images of DMSO- or CB-treated brain mitochondria after immunofluorescence staining with antibodies against cytochrome c_1_ (green) and cyt c (red). Arrows indicate the contact sites of cyt c with cytochrome c_1_.

The results indicating the reduction in CIV-bound cyt c (Fig. 3A), with no significant reduction in total mitochondrial cyt c (Fig. 3B), prompted us to examine the interaction between cyt c and CIII, from which electrons are transported to CIV by cyt c. Immunoblot analysis of the immunoprecipitates of UQCRC2, a core subunit of CIII, revealed that cyt c was coprecipitated with UQCRC2, and the amount of UQCRC2-bound cyt c was increased significantly after treatment with CB (Fig. 4A). Immunoblot analysis was performed to exclude the possibility that the increased amount of UQCRC2-bound cyt c resulting from treatment with CB is due to the increased amount of cytochrome c_1_, a subunit of CIII, which is exposed to IMS and interacts directly with cyt c^23,24^. Cytochrome c_1_ was coprecipitated with UQCRC2, and the amount of UQCRC2-associated cytochrome c_1_ remained unchanged by treatment of the mitochondria with CB (Fig. 4A). Moreover, the total amounts of UQCRC2 and cytochrome c_1_ in the mitochondria were comparable between the control and CB-treated mitochondria (Fig. 4B). These results suggest that the increase in the amount of UQCRC2-bound cyt c induced by CB was not due to the increased amount of cytochrome c_1_ in CIII.

Regarding the association of cyt c with cytochrome c_1_, immunofluorescence staining demonstrated that a portion of cyt c contacted cytochrome c_1_, and the occasional contacts appeared unchanged after treatment of the mitochondria with CB (Fig. 4C).

### Inhibiting actin polymerization enhances the total CcO activity of CIV with no significant alteration in the SC assembly

**Figure 5:**
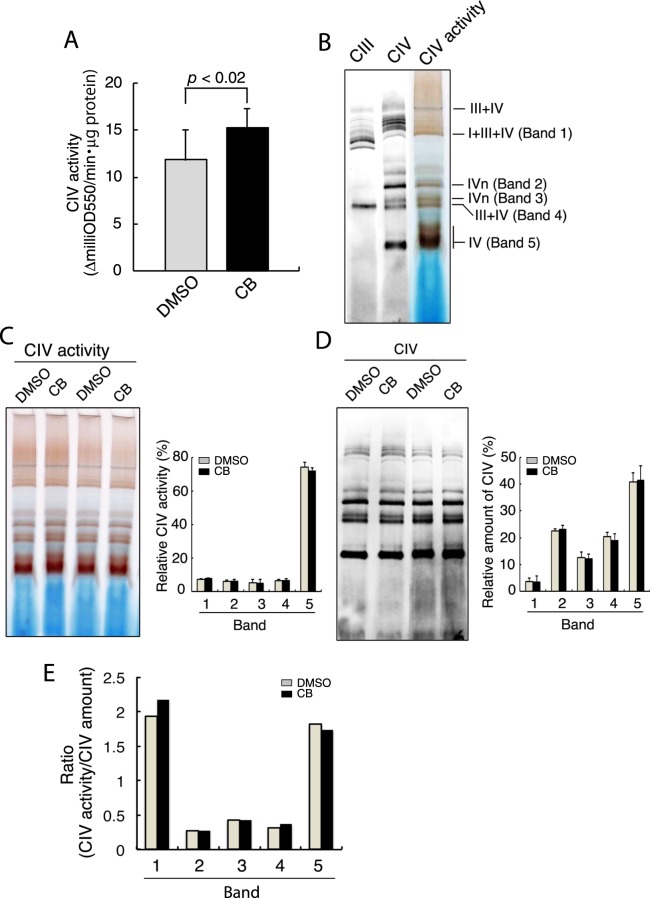
Inhibiting actin polymerization enhances the CcO activity of CIV, with no significant alteration in the SC assembly. (**A**) Spectrophotometric measurement of CcO activity in isolated brain mitochondria after incubation with DMSO or CB for 30 min (n = 10, mean ± S.D.). *p* < 0.02. (**B**) Digitonin mitochondrial lysates were subjected to BN-PAGE and then immunoblotted for CIII (UQCRC2) and CIV (MTCO1) or assayed for the in-gel CcO activity of CIV. The bands corresponding to the SCs, CIV oligomers, and CIV monomers, are indicated. (**C**) In-gel CIV activities were assayed after the BN-PAGE of mitochondrial lysates treated with DMSO or CB. The relative activities of major bands were determined (n = 6, mean ± S.D.). (**D**) The intensities of the CIV bands were measured, and the relative amounts of individual bands were determined (n = 6, mean ± S.D.). (**E**) The relative ratios of the CIV activity to corresponding CIV bands were determined (n = 6, mean ± S.D.).

To examine whether the modified retention of cyt c between CIV and CIII induced by inhibiting actin polymerization with CB was accompanied by enhancement of the CcO activity of CIV, isolated brain mitochondria were incubated with reduced cyt c as the substrate. A spectrophotometric assay revealed that the CcO activity of the mitochondria was significantly enhanced after treatment with CB (Fig. 5A). Respiratory complexes are assembled into supramolecular structures called SCs^9,10^, and the assembly of SCs is thought to determine the respiratory efficiency^25^. Therefore, we next examined SC assembly in isolated brain mitochondria using blue native PAGE (BN-PAGE)^26,27^, followed by an in-gel assay for the CcO activity of CIV^28^. Major bands with CcO activity were labeled according to the previously described nomenclature^8,10,29^ after immunoblotting for CIV (MTCO1) and CIII (UQCRC2). BN-PAGE analysis of the digitonin lysates of the mitochondria revealed that the major CcO activity was associated with band 1 (I + III + IV), bands 2 and 3 (IV oligomers), band 4 (III + IV), and band 5 (IV monomers) (Fig. 5B). Quantification of the band intensities of the CcO activity indicated that more than 70% of the CcO activity was associated with the band 5 CIV monomers (Fig. 5C). In addition, CB treatment of the isolated mitochondria did not alter the individual CcO activities associated with the five corresponding bands (Fig. 5C). In contrast, immunoblot analysis demonstrated that only 10 to 20% of CIV was detected in bands 2, 3 and 4, but approximately 40% of CIV was in the CIV monomers (Fig. 5D), indicating an uneven distribution of the activity and amount of CIV among the bands. Determination of the relative ratio of the CcO activity to the amount of corresponding CIV revealed that the ratio was more than 1.5 for bands 1 and 5, and smaller than 0.5 for bands 2, 3 and 4 (Fig. 5E), suggesting that the SC (I + III + IV) and CIV monomers had higher specific CcO activities than did the SC (III + IV) and CIV oligomers. However, these differential ratios among the bands remained unchanged after treatment of the mitochondria with CB (Fig. 5E).

## Discussion

Immunoprecipitation and immunoblot analyses demonstrated that actin was coprecipitated with MTCO1, a subunit of CIV, indicating the association of actin with CIV. It has been established that actin filaments are involved in the process of mitochondrial fission and localized outside mitochondria^12–17^. In contrast, little is known about actin localization inside mitochondria^18,19^. In the current study, the localization of actin filaments inside brain mitochondria was clearly indicated by the extreme proteolytic digestion and immunofluorescence staining of the mitochondria. Most of the OMM proteins VDAC1/porin and Tom20 were removed; however, most of the IMS protein cyt c, IMM protein MTCO1, mitochondrial matrix protein Hsp60, and actin, remained intact after the digestion of isolated brain mitochondria with 40 to 80 mg/ml trypsin. As actin filaments are associated with mitochondrial DNA in the mitochondrial matrix^18,19^, the present results suggest that mitochondrial actin is localized in the mitochondrial matrix and associated with the N- and C-terminal regions of MTCO1, which are exposed to the mitochondrial matrix^21^. This assumption is supported by the results showing actin filaments localization inside of the OMM protein Tom20 and a portion of the actin filaments contacting the IMM protein MTCO1.

When the actin polymerization inhibitor CB^20^ was used to elucidate the possible involvement of actin filaments in the regulation of mitochondrial function, the OCR of CIV in isolated brain mitochondria was significantly enhanced, suggesting the involvement of actin filaments in the regulation of the OCR. This effect of CB on the OCR in isolated brain mitochondria is similar to that of water-solubilized exogenous CoQ_10_^8^. However, the CB treatment did not increase the amount of MTCO1-associated L-Opa1, indicating a difference between the mechanisms of action of CB and CoQ_10_ in enhancing the OCR in brain mitochondria. The oxidation of cyt c to produce protons mediated by the CcO of CIV is the initial reaction prior to oxygen consumption. Spectrophotometric measurement of the CcO activity in isolated mitochondria revealed that the activity was significantly enhanced by treatment with CB. This finding is in accordance with the result indicating the enhancement of the OCR induced by CB. Taken together, the results suggest that the actin filaments localized within brain mitochondria play a crucial role in the regulation of the CcO activity and OCR of CIV.

Regarding the reason for the enhancement of the CcO activity and OCR induced by CB, immunoblot analyses indicated a significant reduction in the amount of MTCO1-bound cyt c induced by CB. Because the total amounts of mitochondrial cyt c and cyt c-interacting MTCO2^21^ remained unchanged after CB treatment, it is suggested that the reduced CIV-bound cyt c induced by CB was not due to the reduced amount of MTCO2 in CIV. On the other hand, the amount of cyt c that was coprecipitated with UQCRC2, a core subunit of CIII, was significantly increased after CB treatment. This result suggests that the amount of CIII-bound cyt c significantly increased by inhibiting actin polymerization. Because the amounts of cyt c and UQCRC2-associated cytochrome c_1_, the interface for cyt c in CIII^23,24^, were comparable before and after treatment of the isolated brain mitochondria with CB, the increased amount of CIII-bound cyt c is not due to an increased amount of cytochrome c_1_ in CIII, but is offset by a decreased amount of CIV-bound cyt c. The enhanced CcO activity and OCR of CIV and the reduced binding of cyt c to CIV after the treatment of isolated mitochondria with CB are suggested to reflect a rapid association and dissociation of the cyt c-CIV complex, which facilitates the sequential activation of the CcO activity of CIV^30^. Moreover, enhancement of the CcO activity and OCR by CB is not suggested to be due to the altered assembly of CIV-containing SCs. The respiratory efficiency is controlled by the assembly of SCs^25^; however, the present results demonstrated that the treatment of isolated mitochondria with CB did not induce any alterations in the composition of the SC assembly or in the CcO activity of individual CIV-containing SCs or CIV monomers.

In conclusion, this work sheds new light on the regulation of mitochondrial function by actin filaments within mitochondria, and we propose a novel function of the mitochondrial actin filaments in the retention of cyt c between CIV and CIII. It is important to further explore the relationship between the modified retention of cyt c between CIV and CIII and the enhancement of CcO activity and the OCR in isolated brain mitochondria.

## Methods

The mice and animal care, mitochondria isolation, immunoprecipitation, immunoblot analysis, antibodies, and other reagents are all described in detail in the Supplemental Experimental Procedures. All the protocols for animal use and experimentation followed the Principles of Laboratory Animal Care (NIH publication No. 86–23, revised 1985), and the Animal Care Committee of the Tokyo Metropolitan Institute of Gerontology reviewed and approved all the study protocols.

### Silver staining and mass spectrometry

After SDS-PAGE of the immunoprecipitates with control IgG and anti-MTCO1 antibody, proteins in the gels were stained with silver using a Pierce Silver Stain kit (#24612; Thermo Scientific) according to the manufacturer’s instructions. Protein bands were manually excised and cut into small pierces. In-gel digestion was performed using the XL-TrypKit (APRO Science), and tryptic peptides were subjected to LC-MALDI-TOF/MS analysis. Samples of duplicate experiments were fractionated and automatically spotted onto MALDI plates using the direct nano-LC and MALDI fraction system (DiNa-MaP, KYA Technologies). Mass spectra were acquired using a TOF/TOF™ 5800 system (SCIEX) operated on TOF/TOF™ Series Explorer™ software version 4.1 (SCIEX). Protein identification using an MS/MS ion search (MIS) was performed using ProteinPilot™ software (ver.4.5; SCIEX). Protein identifications were considered to be correct based on the following selection criteria: a protein score (ProtScore Unused) higher than 1.3, and at least two peptides with an ion score above 95% confidence.

### Immunofluorescence confocal microscopy

For mitochondrial staining, the mitochondrial suspension in 0.25 M sucrose-containing mitochondrial homogenizing buffer was fixed with 10 volumes of 20% (w/v) phosphate-buffered formalin solution (Wako) and dried on slide glass. The specific primary antibodies were against actin (A5541; Sigma), cytochrome c_1_ (GTX101017, GeneTex), MTCO2 (55070-1-AP; Proteintech), Tom20 (ab186735), MTCO1 (ab203912), and cytochrome c (ab110325) and were obtained from Abcam. The secondary antibodies were Alexa Fluor 488-conjugated anti-rabbit IgG (A11070) and Alexa Fluor 555-conjugated anti-mouse IgG (A21425) and were obtained from Invitrogen. Specimens immunostained with different antibodies were examined using the TCS SP8X confocal microscopy system (Leica).

### Measurement of the OCR

The OCR of isolated mitochondria (100~300 µg of protein) in 2 ml of mitochondrial respiration medium MO5 (20 mM HEPES, 110 mM sucrose, 0.5 mM EGTA, 3 mM MgCl_2_, 60 mM K-lactobionate, 20 mM taurine, 10 mM KH_2_PO_4_, 10 μM cytochrome c, and 1 g/l BSA; pH 7.1) was determined at 37 °C using high-resolution respirometry (Oxygraph-2k, Oroboros). Complex I respiration was specifically assessed through the addition of 2.5 mM ADP in the presence of 5 mM malate and 10 mM glutamate^5,8^. In certain experiments, 20 μM cytochalasin B (ab143482; Abcam) dissolved in DMSO was added to MO5 and incubated at 37 °C for 30 min for further analysis.

### Spectrophotometric assay for the CcO activity of CIV

The CcO activity of CIV was determined by measuring the reduced form of cyt c. Immediately after mixing the mitochondrial fraction prepared from the fresh brain samples in the mitochondria homogenizing buffer with reduced cyt c (ab140219, Abcam) in a 96-well plate, the absorbance at 550 nm and 30 °C was monitored using a spectrophotometer (Perkin Elmer). An aliquot of the mitochondrial fraction was used to determine the protein content with a protein reagent kit (Thermo Scientific). The specific activity was calculated as ∆milliOD550 per min·μg protein.

### BN-PAGE and in-gel CIV activity assay

The mitochondrial fraction was solubilized with 5% (w/v) digitonin (Invitrogen)^31,32^ for 15 min on ice. After centrifugation at 20,000 × g for 30 min at 4 °C, the clear supernatant was loaded onto 3–12% BN gels (Invitrogen). For the immunoblot analysis, proteins in the gels were transferred onto a PVDF-FL membrane (Merck). The in-gel CcO activity of CIV was visualized by the precipitation of 3, 3′-diaminobenzidine oxides and indamine polymers in test buffer containing 50 mM sodium phosphate, 0.5 mg/ml 3, 3′-diaminobenzidine-tetrahydrochloride (D5905; Sigma), 0.25 mg/l cyt c (C2037; Sigma), and 75 mg/ml sucrose, pH 7.4^32^.

### Statistical analysis

Unpaired Student’s t-tests were applied to evaluate differences between experimental groups. The standard deviation of the mean (S.D.) was calculated for the results, and *p*-values less than 0.05 were considered to indicate statistical significance.

## Electronic supplementary material

Supplementary information
