# Supplementary material for: In vitro rejuvenation of brain mitochondria by the inhibition of actin polymerization
Source: Sci Rep. 2018 Oct 22;8:15585. doi: 10.1038/s41598-018-34006-5 (PMC6197264; doi:10.1038/s41598-018-34006-5)
Supplement: Supplementary file 1 — Supplementary information [file 41598_2018_34006_MOESM1_ESM.pdf]

Supplementary information

In vitro rejuvenation of brain mitochondria by the inhibition of actin polymerization

Kazuhide Takahashi[1], Yuri Miura[2], Ikuroh Ohsawa[1], Takuji Shirasawa[3] & Mayumi Takahashi[1*]

[1]Biological Process of Aging, [2]Proteome Research, Tokyo Metropolitan Institute of Gerontology, Itabashi, Tokyo 173-0015, Japan. [3]Shirasawa Anti-Aging Medical Institute, Bunkyo, Tokyo 113-0033, Japan

Supplemental Experimental Procedures

Mice and animal care. C57BL/6NCr male mice were housed under pathogen-free barrier conditions with a 12-hr dark/light cycle and were fed a standard chow *ad libitum*. All the protocols for animal use and experimentation followed the Principles of Laboratory Animal Care (NIH publication No. 86-23, revised 1985), and the Animal Care Committee of the Tokyo Metropolitan Institute of Gerontology reviewed and approved all the study protocols.

Mitochondria isolation. Brains were homogenized with a Dounce homogenizer (Wheaton) on ice in 20 volumes of a homogenizing buffer (0.25 M sucrose, 10 mM Tris-HCl; pH 7.4, 1 mM EDTA, and 1 mM PMSF). The homogenates were centrifuged at 800 × g for 10 min at 4ºC, and the supernatants were centrifuged at 12,000 × g for 10 min at 4ºC. The two centrifugation steps were repeated once more to yield a pellet as the mitochondrial fraction. Whole mitochondrial lysates were prepared by homogenizing the mitochondrial fraction in a lysis buffer (1% SDS, 1 mM EDTA, 1 mM PMSF, and 10 mM Tris-HCl; pH 7.4). Aliquots of the mitochondrial fractions were used to measure the protein content using a protein reagent kit (Thermo Scientific).

Immunoprecipitation and immunoblot analysis. Proteins in RIPA buffer (10 mM Tris-HCl, pH 7.4, 0.15 M NaCl, 1% sodium deoxycholate, 1% Triton X-100, 0.1% SDS, and 1 mM PMSF) were immunoprecipitated with the indicated primary antibodies and Dynabeads™ Protein A (Invitrogen, Thermo Scientific). The antibodies used for immunoprecipitation were against MTCO1 (ab14705; Abcam) and UQCRC2 (GTX 114873; GeneTex). Whole mitochondrial lysates or immunoprecipitates were separated by SDS-PAGE. After electrophoresis, the proteins were transferred to a PVDF-FL membrane (Merck) via electrophoresis, and the membrane was blocked with blocking buffer. The proteins were detected with the indicated primary antibodies and visualized using IRDye 800CW-conjugated secondary antibodies (Li-Cor) and an infrared imaging system (Odyssey, Li-Cor). The primary antibodies used were against actin (A5441; Sigma), Opa1 (#612602; BD Biosciences), cytochrome c_1 (GTX101717; GeneTex), VDAC1/porin (10866-1-AP; Proteintech), MTCO1 (ab14705), UQCRC2 (ab14745),Tom20 (ab186735), Hsp60 (ab137706), and cyt c (ab110325) from Abcam. The band intensity was quantified using software equipped with the imaging system or

ImageJ software (National Institutes of Health).

Table S1. Mass spectrometry of the 42 kDa protein

N	Protein score	Accession	Name	Species	Peptides (95%)
1	65.02	P60710	Actin, cytoplasmic 1	MOUSE	51
2	6.83	P62737	Actin, aortic smooth muscle	MOUSE	29
3	2.9	Q6IFX2	Keratin, type I cytoskeletal 42	MOUSE	2
4	2	P63260	Actin, cytoplasmic 2	MOUSE	50
